# Low-tube-voltage selection for triple-rule-out CTA: relation to patient size

**DOI:** 10.1007/s00330-016-4607-9

**Published:** 2016-09-29

**Authors:** Richard A. P. Takx, Radko Krissak, Christian Fink, Valentin Bachmann, Thomas Henzler, Mathias Meyer, John W. Nance, Stefan O. Schoenberg, Paul Apfaltrer

**Affiliations:** 1 Department of Radiology, University Medical Center Utrecht, Heidelberglaan 100, P.O. Box 85500, 3584 CX Utrecht, The Netherlands; 2 Institute of Clinical Radiology and Nuclear Medicine, University Medical Center Mannheim, Medical Faculty Mannheim – Heidelberg University, Heidelberg, Germany; 3 Department of Diagnostic and Interventional Radiology, Hufeland Klinikum GmbH, Bad Langensalza, Germany; 4 Department of Radiology, General Hospital Celle, Celle, Germany; 5 Division of Cardiovascular Imaging, Department of Radiology and Radiological Science, Medical University of South Carolina, Charleston, SC USA; 6 Department of Biomedical Imaging and Image-guided Therapy, Medical University of Vienna, Vienna, Austria

**Keywords:** Multidetector computed tomography, Radiation dosage, Image enhancement, Body mass index, Chest pain

## Abstract

**Objectives:**

To investigate the relationship between image quality and patient size at 100 kilovoltage (kV) compared to 120 kV ECG-gated Triple-Rule-Out CT angiography (TRO-CTA).

**Methods:**

We retrospectively included 73 patients (age 64 ± 14 years) who underwent retrospective ECG-gated chest CTA. 40 patients were scanned with 100 kV while 33 patients with 120 kV. Body mass index (BMI), patients’ chest circumference (PC) and thoracic surface area (TSA) were recorded. Quantitative image quality was assessed as vascular attenuation in the ascending aorta (AA), pulmonary trunk (PA) and left coronary artery (LCA) and the signal-to-noise ratio (SNR) in the AA.

**Results:**

There was no significant difference in BMI (26.0 ± 4.6 vs. 28.0 ± 6.7 kg/m^2^), PC (103 ± 7 vs. 104 ± 10 cm^2^) and TSA (92 ± 15 vs. 91 ± 19 cm^2^) between 100 kV and 120 kV group. Mean vascular attenuation was significantly higher in the 100 kV compared to the 120 kV group (AA 438 vs. 354 HU, PA 460 vs. 349 HU, LCA 370 vs. 299 HU all *p* < 0.001). SNR was not significantly different, even after adjusting for patient size. Radiation dose was significantly lower in the 100 kV group (10.7 ± 4.1 vs. 20.7 ± 10.7 mSv; *p* < 0.001).

**Conclusions:**

100 kV TRO-CTA is feasible in normal-to-overweight patients while maintaining image quality and achieving substantial dose reduction.

***Key Points*:**

• *100 kV protocols result in a significantly lower radiation dose.*

• *Mean vascular attenuation is significantly higher using 100 kV.*

• *SNR and CNR are not significantly different between 100 kV and 120 kV.*

• *100 kV CTA is feasible regardless of patient size while maintaining image quality.*

## Introduction

ECG-gated computed tomography angiography (CTA) of the chest has been shown to be a reliable and comprehensive non-invasive imaging test to exclude acute coronary syndrome, aortic dissection and pulmonary embolism in patients presenting to the emergency department with acute chest pain (i.e., Triple Rule Out (TRO)-CTA) [1–9].

However, the potential harm of radiation exposure is of concern, as it has been postulated to be associated with a non-negligible lifetime attributable risk of cancer [10]. This risk varies noticeably and is substantially greater for females, younger patients, and for combined cardiac and aortic scans [10]. Previous studies demonstrated that lowering the kilovoltage (kV) setting from standard 120 kV to 100 kV allows a significant reduction of radiation exposure independent from the CT scanner hardware [11–14]. Radiation dose reduction through kV adjustment, however, may adversely affect image quality due to the inverse relationship between radiation exposure and image noise [15]. Empiric selection criteria for application of reduced peak tube potential include markers of body size (e.g., body mass index [BMI]); however, body size selection criteria are likely crude given interpatient differences in weight distribution and body composition [15]. Patients’ chest area and BMI have been found to frequently be discordant, which can lead to over radiating patients when using BMI to select tube potential [16]. However, there remains hesitation among clinicians to lower kV out of fear for not achieving adequate image quality for diagnosis. The hypothesis of this study was that 100 kV CTA is feasible in normal-to-overweight patients while maintaining appropriate diagnostic image quality. Accordingly, we investigated the relationship between image quality and patient size in patients undergoing 100 kV vs. 120 kV ECG-gated TRO-CTA.

## Materials and methods

### Patient population

Seventy-three consecutive patients (mean age 64 ± 13, range 25–83 years) were retrospectively enrolled. All patients underwent TRO-CTA for acute chest pain evaluation referred by our chest pain unit between October 2009 and January 2011. The 100 kV scanning was performed at the discretion of the attending radiologist. In our institution, TRO-CTA is used to assess patients with acute chest pain for pulmonary embolism, acute aortic syndrome, acute coronary syndrome and other thoracic pathologic conditions with a single examination.

### CT technique

All imaging exams were conducted on a 64-channel dual-source CT scanner (Somatom Definition, Siemens Healthcare, Forchheim, Germany). Retrospectively ECG-gated spiral CTA of the heart was acquired from the level of the carina. Then, 120 ml of iodine contrast (Imeron 400, Bracco Imaging S.p.A., Milan, Italy) was injected using a power injector with an injection rate of 4 ml/s and followed by a saline chaser of 40 ml. Patients were instructed to hold their breath at a mid-inspiratory position. The cranio-caudal acquisition was started using automatic bolus triggering at 2 sec intervals in the ascending aorta (trigger level 100 HU) with a delay of 5 sec. The following additional scan parameters were applied for the TRO-CTA: detector collimation 2 × 32 × 0.6 mm, slice acquisition 2 × 64 × 0.6 mm using z-flying focal spot, gantry rotation time 330 ms, tube current time product 320 mAs, and the pitch automatically adapted to the patient’s heart rate using a reference value of 0.28. Automatic tube current modulation (CARE Dose 4D) and automatic ECG-pulsing for radiation dose reduction were applied. For data reconstruction, axial images of the thoracic scan range were reconstructed with 1.0 mm slice thickness and 0.8 mm increment using a B30f soft tissue kernel. In addition, the heart was reconstructed with a slice thickness of 0.6 mm and 0.5 mm increment using a B26f soft tissue kernel in the optimal diastolic and systolic phase as determined by the CT software. Dose length product (DLP) and volume CT dose index (CTDI_vol_) were collected. The effective dose was calculated using a conversion coefficient of 0.017 mSv mGy^-1^cm^-1^ [17].

### Image analysis

**Fig. 1:**
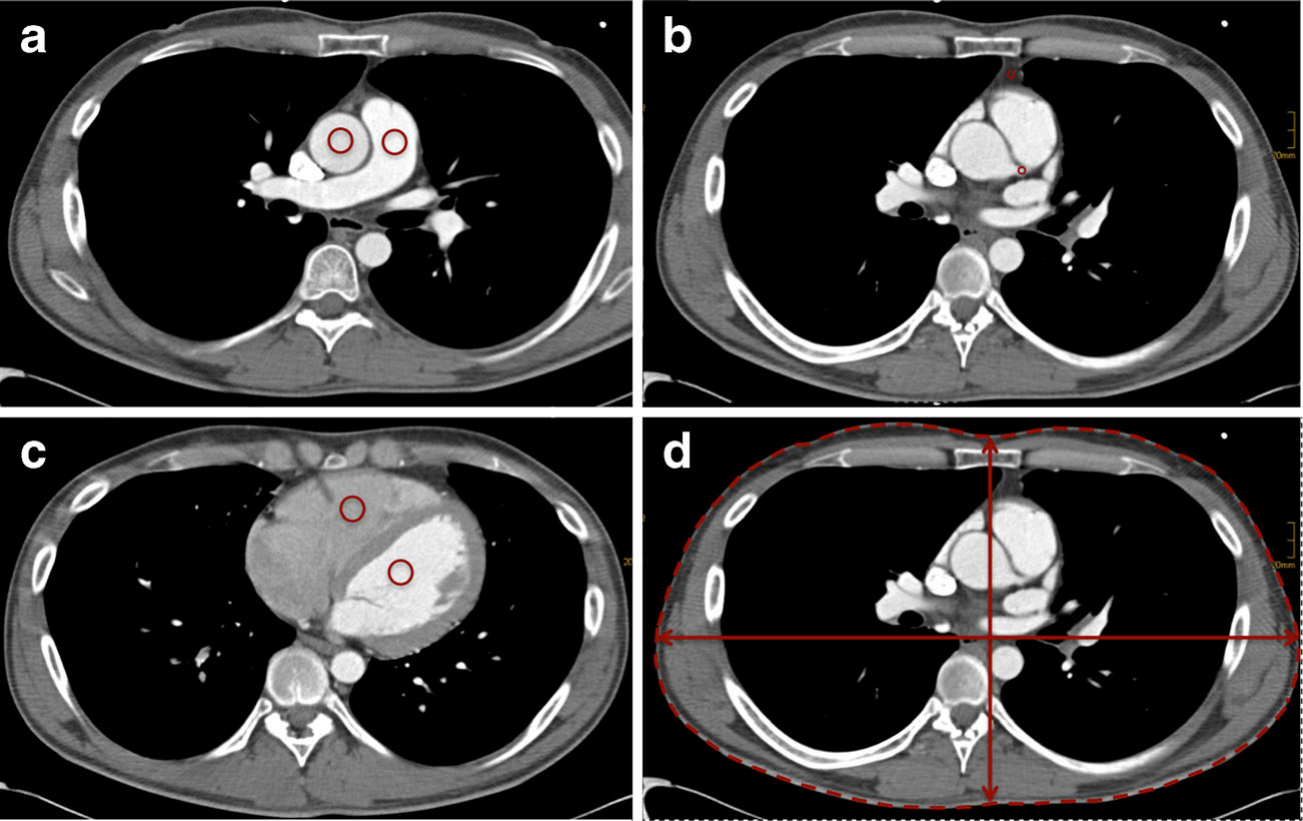
Example image of measurements (**a**) ROI in ascending aorta and pulmonary artery. **b** Left main coronary artery and epicardial fat. **c** left and right ventricle. **d** anthropomorphic measures at level of the origin of the left main coronary artery

The quantitative measurements were performed on the axial images with 1 mm slice thickness by one experienced reviewer (radiologist with 5 years of experience in cardiothoracic imaging). A circular region of interest (ROI) was placed in the following anatomical regions: main pulmonary artery, ascending aorta, left ventricle, right ventricle, left main coronary artery, and epicardial fat. Calcifications and plaques within the ROIs were avoided. Mean attenuation values were recorded in Hounsfield Units (HU). The signal-to-noise ratio (SNR) was defined as the ratio of the mean attenuation in the ascending aorta to the standard deviation of this ROI. Contrast-to-noise ratio (CNR) was defined as the ratio of the mean attenuation in the ascending aorta minus mean attenuation of epicardial fat to the standard deviation of the attenuation value in the ascending aorta. Patient size was measured as BMI, patients’ chest circumference (PC) at the level of the origin of the left main coronary and thoracic surface area (TSA), defined as the product of dorso-ventral and latero-lateral diameters of the chest at the same level as above (Fig. 1).

### Statistical analysis

Continuous variables were listed as mean plus standard deviation (SD). Student’s t-test was used to determine statistical significant differences in mean values. Linear regression was applied to generate adjusted *p*-values. Correlations were calculated using Pearson correlation coefficient or Spearman’s rank. Discrete variables were evaluated using the Fisher’s exact test. A *p*-value smaller than 0.05 was used to indicate statistical significance. Analyses were performed using SPSS (IBM Corp, Version 23.0. Armonk, NY, USA).

## Results

### Baseline characteristics

**Fig. 2:**
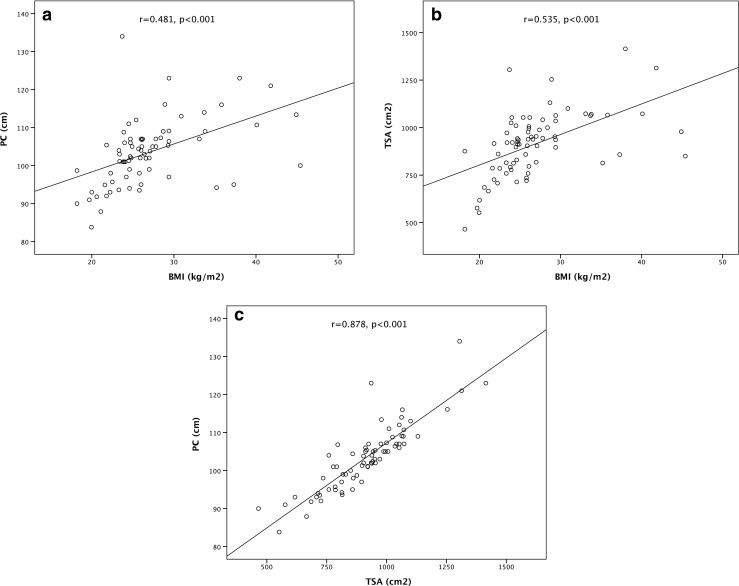
Correlation plot between the various anthropomorphic measures. **a** PC and BMI, **b** TSA and BMI, and **c** PC and TSA

There was no significant difference in BMI between the 100 kV and 120 kV group (26.0 ± 4.6 vs. 28.0 ± 6.7 kg/m^2^; *p* = 0.138), PC (103 ± 7 vs. 104 ± 10 cm^2^; *p* = 0.737) or TSA (92.4 ± 15.2 vs. 90.9 ± 19.2 cm^2^; *p* = 0.815). Both groups showed similar age (65 ± 12 vs. 63 ± 16 years; *p* = 0.521) and similar proportion of males (27 vs. 20 males, *p* = 0.540). Detailed characteristics of the study population are listed in Table 1. The correlation between BMI and TSA/PC was moderate while the correlation between TSA and PC was high (Fig. 2).

**Table 1:** Patient characteristics and radiation dose

	100 kV (*n* = 40)	120 kV (*n* = 33)	*p-*value
BMI (kg/m^2^)	26.0 ± 4.6	28.0 ± 6.7	0.138
<25	19	14	
25.1 – 30	17	11	
>30.1	4	8	
CTDI (mGy)	19.2 ± 6.9	41.3 ± 21.5	<0.001
mAs	162.4 ± 55.6	192.9 ± 70.8	0.044
DLP (mGy cm)	630 ± 240	1215 ± 626	<0.001

### Image quality

**Fig. 3:**
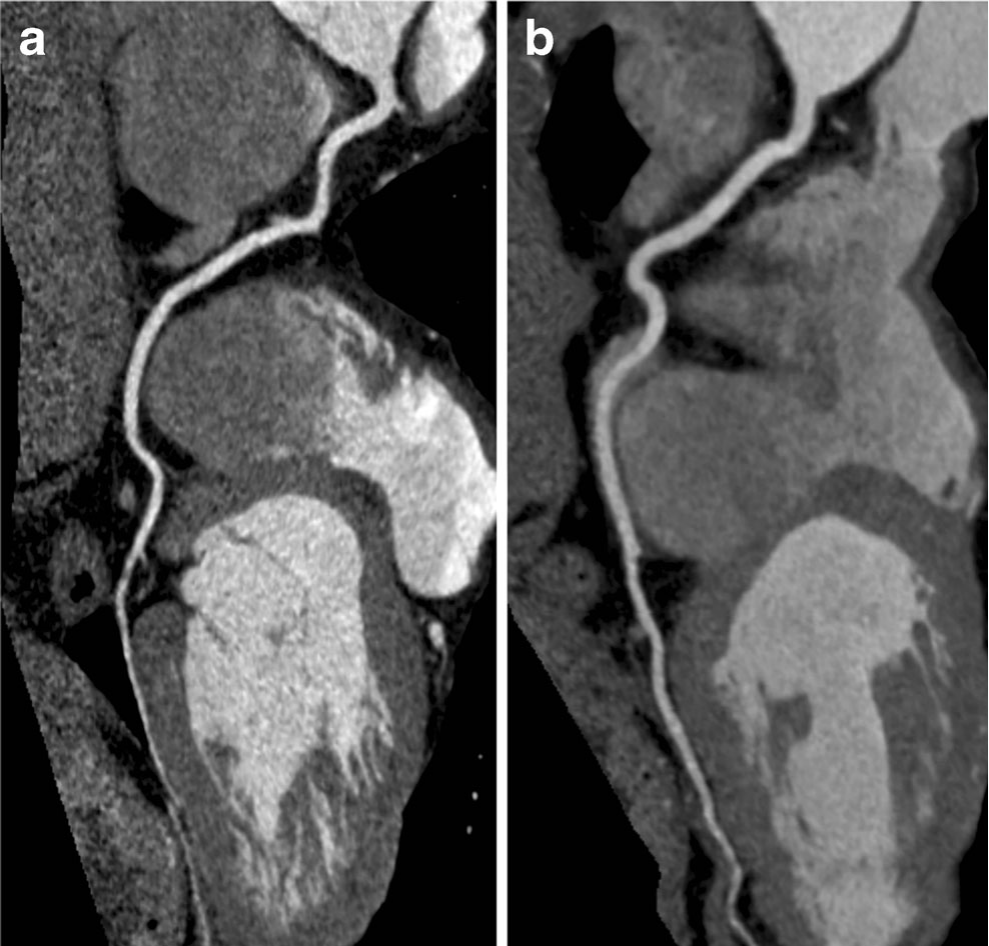
The right coronary artery in patients of similar BMI scanned with Triple-Rule-Out CT angiography at 100 kV (**a**) and 120 kV (**b**)

A radiologist (5 years of experience) graded all studies as of diagnostic quality. The average attenuation of contrast medium in the low-dose group was significantly higher at all vascular levels (all *p* < 0.05; Table 2; Fig. 3). There was no significant difference in the mean attenuation values of epicardial fat between the groups (*p* = 0.40). Image noise in the main pulmonary artery and left main coronary artery was higher in the low-voltage group (*p* < 0.05). Image noise in the right ventricle and epicardial fat showed no significant difference as presented in Table 2.

**Table 2:** Mean attenuation and noise

	100 kV (*n* = 40)	120 kV (*n* = 33)	*p*-value	p-value adjusted for BMI	p-value adjusted for PC	p-value adjusted for TSA
Attenuation						
Pulmonary artery	459.8 ± 107.2	348.6 ± 85.3	<0.001	<0.001	<0.001	<0.001
Ascending Aorta	435.7 ± 102.4	353.6 ± 66.9	<0.001	<0.001	<0.001	<0.001
Left ventricle	407.9 ± 97.6	326.2 ± 68.9	<0.001	<0.001	<0.001	<0.001
Right ventricle	405.5 ± 135.3	335.3 ± 118.9	0.024	0.040	0.029	0.021
Left coronary artery	369.5 ± 117.1	299.4 ± 101.3	0.009	0.015	0.011	0.009
Epicardial fat	99.8 ± 25.5	93.9 ± 25.6	0.335	0.404	0.367	0.336
Noise						
Pulmonary artery	30.3 ± 10.9	22.3 ± 8.5	0.001	<0.001	<0.001	0.001
Ascending Aorta	27.4 ± 9.2	22.3 ± 9.1	0.057	0.061	0.049	0.060
Left ventricle	36.6 ± 15.7	31.9 ± 22.9	0.302	0.188	0.212	0.269
Right ventricle	46.3 ± 21.7	40.8 ± 24.7	0.315	0.231	0.258	0.303
Left main coronary artery	67.9 ± 28.5	48.3 ± 21.9	0.002	0.001	0.002	0.002
Epicardial fat	33.9 ± 14.3	32.0 ± 20.0	0.649	0.657	0.594	0.645
CNR aorta	12.6 ± 4.7	13.8 ± 8.9	0.463	0.419	0.391	0.453
SNR aorta	16.9 ± 6.9	17.7 ± 6.9	0.623	0.460	0.529	0.666

The difference in the calculated SNR and CNR between the two groups was not statistically significant (*p* = 0.46 and *p* = 0.62), which was maintained after adjusting for body size measures.

### Radiation dose

Mean CTDI_vol_ in the 100 kV group was 19.2 ± 6.9 mGy and was significantly lower compared to the 120 kV group (41.3 ± 21.5 mGy, *p* < 0.001). Mean DLP in the 100 kV group was 630 ± 240 mGy cm and was significantly lower in comparison with the 120 kV group (1215 ± 626 mGy cm, *p* < 0.001). The mean estimated effective radiation dose for the 100 kV group was 10.7 ± 4.1 mSv and 20.7 ± 10.7 mSv (*p* < 0.001), corresponding to a 55 % reduction in radiation exposure by lowering tube voltage to 100 kV. Radiation dose results are summarized in Table 1. A moderate correlation was found between BMI and effective radiation dose for both the 100 kV group and the 120 kV group (*r* = 0.326, *p* = 0.043 and *r* = 0.357, *p* = 0.041, respectively).

## Discussion

Our results show that 100 kV TRO-CTA of the chest can be performed with appropriate diagnostic image quality and substantial dose reduction in our patient population, which included predominantly normal-to-overweight individuals. Even after adjusting for measures of patient size, no significant differences were found in image quality between 100 kV and 120 kV. Reducing tube voltage to 100 kV results in an approximately 55 % lower effective radiation dose compared to a 120 kV TRO-CTA protocol performed while maintaining diagnostic image quality. Similar reductions were reported by Feuchtner et al. [12], where a radiation dose reduction of 47 % was achieved using 100 kV.

The mean attenuation values of the pulmonary artery, the ascending aorta, the left ventricle, the right ventricle and the left main coronary artery were significantly higher at 100 kV, which is in line with published data on low kV imaging [18, 19].

This observation can be explained by the photoelectric effect, which results in increased photon attenuation as tube voltages approach the k-edge of a given element; in case of iodine-based contrast materials, vascular attenuation is expected to increase with decreasing tube voltages to approximately 33 kV (the approximate k-edge of iodine) [20, 21]. The photoelectric effect is the dominant mechanism of photon attenuation in materials containing high-atomic number elements (e.g., iodinated contrast material). In contrast, Compton scatter is the dominant interaction in tissues lacking high-atomic number elements (e.g., soft tissue and fat). Accordingly, no significant difference in mean attenuation of the (fatty) tissue adjacent to the left coronary artery was observed [22]. A disadvantage of lower tube voltage (and the major reason for diagnostic tube voltage ranges used in current clinical imaging) is the concomitant increase in image noise [20]. While the noise level for the 100 kV CTA protocol was found to be higher in the current study, as expected, all imaging studies were considered of diagnostic quality, which was supported by non-significant differences in SNR and CNR between both protocols. In a prior study on 100 kV imaging by Krissak et al. [22] the feasibility of TRO-CTA with 100 kV was demonstrated in non-obese patients. In this current study we extend the use of 100 kV imaging to normal-to-overweight patients, which was maintained even after adjusting for measures of patient size.

Despite growing evidence of non-inferior image quality using low kV protocols in nonobese patients [11, 22], data regarding the impact of body habitus, i.e., chest dimensions, PC, and TSA has been lacking. This is of particular concern, as a recent anthropomorphic phantom study found that patients’ chest area and BMI classes were frequently discordant, which could potentially lead to over-radiating patients when using BMI to select the tube voltage [16]. BMI, while an imperfect but convenient proxy for human body fat, does not reliably represent the body shape, especially in patients with central obesity; this can become particularly troublesome in TRO-CTA, where the scan field is limited to the chest. Conversely, a large-chested woman probably requires more radiation than a small-chested man with a similar BMI [23]. Thus, the findings of our study extend prior data, establishing radiation dose reduction and maintaining image quality even after adjusting for patient size.

Our study is limited by its retrospective nature. Second, we only had a small percentage of subjects with a BMI above 30, which may hamper the generalizability of the results to morbidly obese individuals. Finally, in this study we did not evaluate the effect of iterative image reconstruction techniques, which would reduce image noise [24, 25].

In conclusion, 100 kV TRO-CTA can be performed in normal-to-overweight patients, while maintaining image quality and achieving substantial dose reductions.

## Abbreviations

BMI: body mass index
CTA: computed tomography angiography
CTDI_vol_: volume CT dose index
DLP: dose length product
kV: kilovoltage
PC: patients’ chest circumference
TRO: triple rule out
TSA: thoracic surface area
